# Virtual reality for autism: unlocking learning and growth

**DOI:** 10.3389/fpsyg.2024.1417717

**Published:** 2024-12-03

**Authors:** Chiara Failla, Paola Chilà, Noemi Vetrano, Germana Doria, Ileana Scarcella, Roberta Minutoli, Alberto Scandurra, Stefania Gismondo, Flavia Marino, Giovanni Pioggia

**Affiliations:** ^1^Institute for Biomedical Research and Innovation (IRIB), National Research Council of Italy (CNR), Messina, Italy; ^2^Department of Human and Society Sciences, University of Enna Kore, Enna, Italy; ^3^Faculty of Psychology, International Telematic University Uninettuno, Rome, Italy; ^4^Department of Cognitive, Psychological Science and Cultural Studies, University of Messina, Enna, Italy

**Keywords:** virtual reality, autism spectrum disorder, technology, learning, sensory stimulation

## Introduction

In the realm of autism spectrum disorder (ASD), the dynamic intersection with cutting-edge technology, particularly Virtual Reality (VR), has emerged as a groundbreaking pathway for both educational and therapeutic interventions. ASD, characterized by difficulties in social communication and repetitive behaviors, presents unique challenges in navigating daily social interactions (American Psychiatric Association, DSM-5 Task Force, 2013). In recent times, there has been a notable increase in the utilization of technology innovations, particularly virtual reality, to address the varied requirements of people with autism spectrum disorders (Burdea and Coiffet, 2017; Glaser and Schmidt, 2022). This emerging field of autism and technology symbiosis holds promise for improving social, cognitive, and linguistic abilities in a tailored and encouraging way. VR stands out as a key player primed for fast expansion in the modern period, where Information and Communication Technology systems (ICTs) are orchestrating disruptive changes across sectors (UNCTAD, 2020). With its roots in computer graphics, virtual reality (VR) produces rich, sensory experiences for users in virtual surroundings. Immersive VR (IVR) systems, such as Head-Mounted Displays (HMDs), have contributed significantly to the emergence of VR, especially in the field of healthcare. The academic community has gradually explored the use of IVR in healthcare, with the goal of improving learning rates and addressing particular issues related to neurodevelopmental disorders like ASD. The advantages of IVR are well matched with the traits of people with ASD, such as their innate love of technology, superior visual recall, and increased sensitivity to visual-spatial information (Glaser and Schmidt, 2022; Schmidt et al., 2021b). By reducing social anxiety and facilitating teaching in realistic, adaptable virtual worlds, this technology promotes a regulated and reproducible learning context (Zhang et al., 2022; Karami et al., 2021; Parsons, 2016). The literature underscores a surge in studies focusing on IVR as a pivotal training and learning intervention for individuals with ASD, highlighting its profound impact on addressing the core symptoms of the disorder (Bozgeyikli et al., 2018; Miller and Bugnariu, 2016; Lorenzo et al., 2019). However, the effectiveness of IVR stands contingent on its integration with a carefully designed learning strategy, emphasizing the need for a holistic approach in leveraging the potential of this transformative technology for individuals on the autism spectrum.

## Virtual reality and autism spectrum disorder

Advances in technology, particularly in the field of virtual reality (VR), have contributed to the rehabilitation of motor, cognitive and social functions in different areas of neurodevelopment (Molteni, 2019). The general goal of VR in a rehabilitation context is to facilitate the exercise of impaired functions, allowing individuals to manage, overcome, reduce or compensate for deficits. A reference by Stramba Badiale (2019) highlights the impact of VR on cognitive performance, underlining its potential as a therapeutic tool and therefore validating the effectiveness of this tool in the rehabilitation context. In the context of ASD, VR has emerged as an effective tool to create authentic virtual environments that improve social skills in a safe environment. VR treatments conducted through individual sessions, have different purposes for children with ASD, which include preparation for public speaking, the development of both simple and complex social skills, the acquisition of stress management techniques and the development of new skills. A particularly advantageous aspect of VR sessions is the characteristic of having a controlled environment in which it is possible to make mistakes, lowering the levels of anxiety perceived and experienced in natural situations of social contexts that are always very unpredictable. The evolution of VR technology supports its integration into educational practices for both children and adults, offering immersive 3D scenarios that can produce the development of adaptive behaviors. The choice of VR technology is fundamental to replicate the characteristics of the real world and structure behaviors. The main strengths of VR include controllability, replicability and its beneficial impact for individuals in experiencing unfamiliar social situations (Lorenzo et al., 2019; Parsons and Mitchell, 2002). It is essential to note that the immersive nature of VR requires individuals to wear specific devices, which may raise concerns about perceived invasiveness, especially among individuals with ASD who in some cases, due to sensory aspects, may poorly tolerate the use of the device and therefore, potentially negatively influence acceptance and willingness to participate in VR-based interventions. All of this highlights the importance of considering the psychological and sensory aspects of technology implementation, especially when working with populations facing unique challenges such as ASD.

## Virtual reality in autism therapy

VR in the field of autism stands out for its ability to offer a safe environment in which subjects can experience social situations and tasks, often difficult to deal with in real life (Mesa-Gresa et al., 2018). Research emphasizes different areas of intervention with a main focus on improving social skills (Ip et al., 2018; Manju et al., 2018; Bekele et al., 2016; Didehbani et al., 2016; Ip et al., 2016; Ke and Lee, 2016) and also shows the use of VR as a tool with good potential in promoting the understanding and management of emotions by facilitating the development of such skills for children with ASD (Chen et al., 2016; Lorenzo et al., 2016; Chen et al., 2015; Lamash et al., 2017). Research also demonstrates how communication represents another crucial area of intervention in which technology offers tools to facilitate both verbal and non-verbal communication (Taryadi, 2018; Parsons, 2015; Cai et al., 2013). At the same time, there is a growing interest in applying virtual reality to help individual with ASD deal with tasks of daily living (Adjorlu et al., 2017; Lamash et al., 2017; Wade et al., 2016). Various studies, mainly using Oculus technology, show the effectiveness of virtual reality in teaching social skills. Examples in the literature include “Bob's Fish Shop,” a VR environment designed for the Oculus Rift headset, aimed at helping children with ASD practice typical social interactions and conversational skills within a virtual store (Schmidt et al., 2021a), as well as studies demonstrating success in teaching safety and sensory regulation skills (Stewart Rosenfield et al., 2019; Dixon et al., 2020). Additionally, VR has been shown to be effective in teaching monetary skills and supporting social skill development through role-play scenarios (Johnston et al., 2020). While the existing literature focuses predominantly on Oculus Rift technology, the potential application of newer platforms such as Oculus Quest remains an area of future exploration. Scientific evidence underscores the success of VR, in conjunction with robotic instrumentation, in improving the social, cognitive, and communication skills of individuals with autism, presenting promising avenues for new modalities of rehabilitative interventions.

## Artificial intelligence and technology in autism therapy

Artificial intelligence (AI) has the potential to significantly contribute to the personalization of assistive devices for people with autism (https://www.verywellhealth.com/assistive-technology-for-autism-5076159, accessed December 15, 2023). Real-time adaptation of assistive technology could be a potential application of artificial intelligence. This means that settings on devices may be automatically changed in response to user activities and interactions. For example, AI can intervene to provide targeted support if a person with autism shows symptoms of stress or dissatisfaction. As shown by Iannone and Giansanti (2024) in a narrative review, the intersection between artificial intelligence and technology can have a great impact on intervention pathways in the field of autism. Over the past 5 years, research has focused on various aspects of autism characteristics using technology-enhanced AI. For example, the use of AI-driven socially assistive robots and smart glasses is proving promising for improving social-emotional behaviors and facilitating school inclusion (Silvera-Tawil et al., 2022; Jain et al., 2020; Vahabzadeh et al., 2018; Keshav et al., 2017). Another type of use of AI and technology concerns the use of advanced machine learning algorithms that are enhancing recommendation systems for sensory management and automatic diagnosis of ASD, offering new tools for therapists, teachers and families (Kumar and Das, 2021).

## Synergy of VR and AI in autism

The convergence of artificial intelligence (AI) and virtual reality (VR) can open transformative paths in autism interventions, providing highly personalized and dynamic therapeutic environments. VR, thanks to its immersive capabilities, allows individual with ASD to practice essential social and daily living skills in interactive and controlled contexts that can help lower the anxiety levels typical of subjects in these situations. Meanwhile, the addition of AI amplifies the adaptability and personalization of these experiences by allowing real-time adjustments in response to the behavior and emotional state of the user, favoring the possibility of carrying out training that can control internal variables of the individual that can usually be an obstacle during the learning phases. Thus, this synergy allows AI to monitor stress indicators and automatically adapt VR environments to reduce anxiety and improve participant engagement by promoting a more supportive learning space and less characterized by unpredictability and the possibility of failure for the subject. The integration of advanced machine learning algorithms also enables VR platforms to provide precise and evidence-based support for therapists, educators and healthcare professionals (https://www.verywellhealth.com/assistive-technology-for-autism-5076159, accessed December 15, 2023) (Iannone and Giansanti, 2024; Silvera-Tawil et al., 2022; Jain et al., 2020; Vahabzadeh et al., 2018; Keshav et al., 2017; Kumar and Das, 2021). The use of AI-driven VR for autism intervention therefore offers new levels of personalization and accessibility in therapeutic approaches.

## Discussion

The VR and AI technologies are emerging as game-changing tools in autism interventions, offering highly customizable and immersive solutions to address the complex social, cognitive and behavioral challenges of individuals with ASD. VR's ability to create safe, controllable and repeatable environments enables targeted learning, reducing anxiety associated with real-world settings and facilitating the generalization of learned skills. Integration with AI further amplifies the effectiveness of these interventions, allowing for dynamic, real-time adaptation to the specific needs of each individual, thanks to features such as speech recognition, behavioral analysis and personalized sensory interaction. Furthermore, technological advances, such as the use of more accessible and mobile platforms, promise to expand access to these resources, overcoming traditional logistical barriers. A crucial element to maximize the effectiveness of these technologies is the development and implementation of new research protocols that ensure scientifically validated and standardized use, without sacrificing the aspect of customization. Structured protocols would help identify best practices, offering clear guidelines for professionals working with individuals with ASD. At the same time, the flexibility to adapt interventions to the unique needs of each person, using artificial intelligence to calibrate virtual experiences in real time, would significantly reduce the risk of error and exposure to external social judgment, which is often a source of discomfort for these individuals. Standardization combined with personalization can help provide more effective support for the development of social, cognitive and communication skills, offering individuals with ASD more structured and safe opportunities to address daily challenges. Creating a balance between standardization and adaptability would allow for more replicable and scientifically robust interventions, opening new avenues for inclusive and high-quality therapeutic approaches.

## References

[B1] Adjorlu A. Høeg E. R. Mangano L. Serafin S. (2017). “Daily living skills training in virtual reality to help children with autism spectrum disorder in a real shopping scenario,” in 2017 IEEE International Symposium on Mixed and Augmented Reality (ISMAR-Adjunct) (Nantes), 294–302. 10.1109/ISMAR-Adjunct.2017.93

[B2] American Psychiatric Association DSM-5 Task Force. (2013). Diagnostic and Statistical Manual of Mental Disorders: DSM-5. Washington, DC: American Psychiatric Association. 10.1176/appi.books.9780890425596

[B3] Bekele E. Wade J. Bian D. Fan J. Swanson A. Warren Z.. (2016). “Multimodal adaptive social interaction in virtual environment (MASI-VR) for children with Autism spectrum disorders (ASD),” in 2016 IEEE Virtual Reality (VR) (Greenville, SC), 121–130. 10.1109/VR.2016.7504695

[B4] Bozgeyikli L. Raij A. Katkoori S. Alqasemi R. (2018). A survey on virtual reality for individuals with autism spectrum disorder: design considerations. IEEE Trans. Learn. Technol. 11, 133–151. 10.1109/TLT.2017.2739747

[B5] Burdea G. C. Coiffet P. (2017). Virtual Reality Technology. Hoboken, NJ: Wiley.

[B6] Cai Y. Chia N. K. Thalmann D. Kee N. K. Zheng J. Thalmann N. M. (2013). Design and development of a virtual dolphinarium for children with autism. IEEE Trans. Neural Syst. Rehabil. Eng. 21, 208–217. 10.1109/TNSRE.2013.2240700 23362251

[B7] Chen C. H. Lee I. J. Lin L. Y. (2015). Augmented reality-based self-facial modeling to promote the emotional expression and social skills of adolescents with autism spectrum disorders. Res. Dev. Disabil. 36, 396–403. 10.1016/j.ridd.2014.10.015 25462499

[B8] Chen C. H. Lee I. J. Lin L. Y. (2016). Augmented reality-based video-modeling storybook of nonverbal facial cues for children with autism spectrum disorder to improve their perceptions and judgments of facial expressions and emotions. Comput. Hum. Behav. 55, 477–485. 10.1016/j.chb.2015.09.033

[B9] Didehbani N. Allen T. Kandalaft M. Krawczyk D. Chapman S. (2016). Virtual reality social cognition training for children with high functioning autism. Comput. Hum. Behav. 62, 703–711. 10.1016/j.chb.2016.04.033 PMC3536992 22570145

[B10] Dixon D. R. Miyake C. J. Nohelty K. Novack M. N. Granpeesheh D. (2020). Evaluation of an immersive virtual reality safety training used to teach pedestrian skills to children with autism spectrum disorder. Behav. Analysis Pract. 13, 631–640. 10.1007/s40617-019-00401-1 32953391 PMC7471232

[B11] Glaser N. Schmidt M. (2022). Systematic literature review of virtual reality intervention design patterns for individuals with autism spectrum disorders. Int. J. Hum.–Comput. Interact. 38, 753–788. 10.1080/10447318.2021.1970433

[B12] Iannone A. Giansanti D. (2024). Breaking barriers—the intersection of AI and assistive technology in autism care: a narrative review. J. Pers. Med. 14:41. 10.3390/jpm14010041 38248742 PMC10817661

[B13] Ip H. H. Wong S. W. Chan D. F. Byrne J. Li C. Yuan V. S.. (2016). “Virtual reality enabled training for social adaptation in inclusive education settings for school-aged children with autism spectrum disorder (ASD),” in Proceedings of the International Conference on Blending Learning, Beijing, China (Cham: Springer). 10.1007/978-3-319-41165-1_9

[B14] Ip H. H. S. Wong S. W. L. Chan D. F. Y. Byrne J. Li C. Yuan V. S. N.. (2018). Enhance emotional and social adaptation skills for children with autism spectrum disorder: a virtual reality enabled approach. Comput. Educ. 117, 1–15. 10.1016/j.compedu.2017.09.010

[B15] Jain S. Thiagarajan B. Shi Z. Clabaugh C. Matarić M. J. (2020). Modeling engagement in long-term, in-home socially assistive robot interventions for children with autism spectrum disorders. Sci. Robot. 5:eaaz3791. 10.1126/scirobotics.aaz3791 33022604

[B16] Johnston D. Egermann H. Kearney G. (2020). SoundFields: a virtual reality game designed to address auditory hypersensitivity in individuals with autism spectrum disorder. Appl. Sci. 10:2996. 10.3390/app10092996

[B17] Karami B. Koushki R. Arabgol F. Rahmani M. Vahabie A.-H. (2021). Effectiveness of virtual/augmented reality–based therapeutic interventions on individuals with autism spectrum disorder: a comprehensive meta-analysis. Front. Psychiatry 12:665326. 10.3389/fpsyt.2021.665326 34248702 PMC8260941

[B18] Ke F. Lee S. (2016). Virtual reality based collaborative design by children with high-functioning autism: design-based flexibility, identity, and norm construction. Interact. Learn. Environ. 24, 1511–1533. 10.1080/10494820.2015.1040421

[B19] Keshav N. U. Salisbury J. P. Vahabzadeh A. Sahin N. T. (2017). Social communication coaching smartglasses: well tolerated in a diverse sample of children and adults with autism. JMIR Mhealth Uhealth 5:e140. 10.2196/mhealth.8534 28935618 PMC5629347

[B20] Kumar C. J. Das P. R. (2021). The diagnosis of ASD using multiple machine learning techniques. Int. J. Dev. Disabil. 68, 973–983. 10.1080/20473869.2021.1933730 36568623 PMC9788716

[B21] Lamash L. Klinger E. Josman N. (2017). “Using a virtual supermarket to promote independent functioning among adolescents with Autism Spectrum Disorder,” in 2017 International Conference on Virtual Rehabilitation (ICVR) (Montreal, QC), 1–7, 10.1109/ICVR.2017.8007467

[B22] Lorenzo G. Lledó A. Arráez-Vera G. Lorenzo-Lledó A. (2019). The application of immersive virtual reality for students with ASD: a review between 1990–2017. Educ. Inf. Technol. 24, 127–151. 10.1007/s10639-018-9766-7

[B23] Lorenzo G. Lledó A. Pomares J. Roig R. (2016). Design and application of an immersive virtual reality system to enhance emotional skills for children with autism spectrum disorders. Comput. Educ. 98, 192–205. 10.1016/j.compedu.2016.03.018

[B24] Manju T. Padmavathi S. Tamilselvi D. (2018). “A rehabilitation therapy for autism spectrum disorder using virtual reality,” in Smart Secure Systems – IoT and Analytics Perspective. ICIIT 2017. Communications in Computer and Information Science, Vol. 808, eds. G. Venkataramani, K. Sankaranarayanan, S. Mukherjee, K. Arputharaj, and S. Sankara Narayanan (Singapore: Springer). 10.1007/978-981-10-7635-0_26

[B25] Mesa-Gresa P. Gil-Gómez H. Lozano-Quilis J. A. Gil-Gómez J. A. (2018). Effectiveness of virtual reality for children and adolescents with autism spectrum disorder: an evidence-based systematic review. Sensors 18:2486. 10.3390/s18082486 30071588 PMC6111797

[B26] Miller H. L. Bugnariu N. L. (2016). Level of immersion in virtual environments impacts the ability to assess and teach social skills in autism spectrum disorder. Cyberpsychol. Behav. Social Netw. 19, 246–256. 10.1089/cyber.2014.0682 26919157 PMC4827274

[B27] Molteni F. (2019). La tecnologia nel processo riabilitativo: indicazioni, limiti e prospettive. MR S 1, S2–S3.

[B28] Parsons S. (2015). Learning to work together: Designing a multi-user virtual reality game for social collaboration and perspective-taking for children with autism. Int. J. Child-Comput. Interact. 6, 28–38. 10.1016/j.ijcci.2015.12.002

[B29] Parsons S. (2016). Authenticityinvirtualrealityforassessmentandintervention in autism: a conceptual review. Educ. Res. Rev. 19, 138–157. 10.1016/j.edurev.2016.08.001

[B30] Parsons S. Mitchell P. (2002). The potential of virtual reality in social skills training for people with autistic spectrum disorders. J. Intell. Disabil. Res. 46, 430–43. 10.1046/j.1365-2788.2002.00425.x 12031025

[B31] Schmidt M. Newbutt N. Schmidt C. Glaser N. (2021a). A process-model for minimizing adverse effects when using head mounted display-based virtual reality for individuals with autism. Front. Virtual Real. 2:611740. 10.3389/frvir.2021.611740

[B32] Schmidt M. Schmidt C. Glaser N. Beck D. Lim M. Palmer H.. (2021b). Evaluation of a spherical video-based virtual reality intervention designed to teach adaptive skills for adults with autism: a preliminary report. Interact. Learn. Environ. 29, 345–364. 10.1080/10494820.2019.1579236

[B33] Silvera-Tawil D. Bruck S. Xiao Y. Bradford D. (2022). Socially-assistive robots to support learning in students on the autism spectrum: investigating educator perspectives and a pilot trial of a mobile platform to remove barriers to implementation. Sensors 22:6125. 10.3390/s22166125 36015887 PMC9416372

[B34] Stewart Rosenfield N. Lamkin K. Re J. Day K. Boyd L. Linstead E. A.. (2019). Virtual reality system for practicing conversation skills for children with autism. Multimodal Technol. Interact. 3:28. 10.3390/mti3020028

[B35] Stramba Badiale M. (2019). Influenza della realtà virtuale sulle prestazioni cognitive. Giornale italiano di Medicina Riabilitativa. MR S1, S18–S19.

[B36] Taryadi and Kurniawan, I.. (2018). The improvement of autism spectrum disorders on children communication ability with PECS method multimedia augmented reality-based. J. Phys. Conf. Ser. 947:012009. 10.1088/1742-6596/947/1/012009

[B37] UNCTAD (2020). The Impact of Rapid Technological Change on Sustainable Development, United Nations Conference on Trade and Development. Geneva: UNCTAD.

[B38] Vahabzadeh A. Keshav N. U. Abdus-Sabur R. Huey K. Liu R. Sahin N. T.. (2018). Improved socio-emotional and behavioral functioning in students with autism following school-based smartglasses intervention: multi-stage feasibility and controlled efficacy study. Behav. Sci. 8:85. 10.3390/bs8100085 30241313 PMC6209889

[B39] Wade J. Zhang L. Bian D. Fan J. Swanson A. Weitlauf A.. (2016). Gaze-contingent adaptive virtual reality driving environment for intervention in individuals with autism spectrum disorders. ACM Trans. Interact. Intell. Syst. 6, 1–23. 10.1145/2892636

[B40] Zhang M. Ding H. Naumceska M. Zhang Y. (2022). Virtual reality technology as an educational and intervention tool for children with autism spectrum disorder: current perspectives and future directions. Behav. Sci. 12:138. 10.3390/bs12050138 35621435 PMC9137951

